# The Dox-pDC - A murine conditionally immortalized plasmacytoid dendritic cell line with native immune profile

**DOI:** 10.1371/journal.pone.0192437

**Published:** 2018-02-28

**Authors:** Sebastian Thieme, Alexander Holzbaur, Ralf Wiedemuth, Aline Binner, Katrin Navratiel, Konstantinos Anastassiadis, Sebastian Brenner, Cornelia Richter

**Affiliations:** 1 Department of Pediatrics, University Clinic ‘Carl Gustav Carus’ Dresden, Dresden, Germany; 2 Stem Cell Engineering, BIOTEC, Technische Universitaet Dresden, Dresden, Germany; 3 Center for Regenerative Therapies Dresden, Technische Universitaet Dresden, Dresden, Germany; University of Medicine and Dentistry of New Jersey - New Jersey Medical School, UNITED STATES

## Abstract

Plasmacytoid dendritic cells (pDC) constitute a very rare blood cell population and play a significant role in immune response and immune-mediated disorders. Investigations on primary pDCs are hindered not only due to their rarity but also because they represent a heterogeneous cell population which is difficult to culture *ex vivo*. We generated a conditionally immortalized pDC line (Dox-pDC) from mice with Doxycycline-inducible SV40 Large T Antigen with a comparable immune profile to primary pDCs. The Dox-pDC secrete pro- and anti-inflammatory cytokines upon Toll-like receptor 9 stimulation and upregulate their MHCI, MHCII and costimulatory molecules. Further, the Dox-pDC activate and polarize naïve T cells in vivo and in vitro in response to the model antigen Ovalbumin. Due to their long-term culture stability and their robust proliferation Dox-pDC represent a reliable alternative to primary mouse pDC.

## Introduction

Detailed investigation of rare immune cells often fails due to their poor availability from primary sources or their stability in cell culture. Plasmacytoid dendritic cells (pDC) represent with < 1% of blood cells a very small population of immune cells.[1] They are specialists in sensing nucleic acids of viral and bacterial origin with their Toll-like receptors (TLR) 7 and 9 and respond to it with huge amounts of type I interferons (IFN) and other pro-inflammatory cytokines.[2, 3] pDC drives both the innate and adaptive immune response. Secreting IFNα, interleukin (IL)-12 and IL-18 pDC activate natural killer cells and conventional dendritic cells.[4–6] TLR-activated pDC express the major histocompatibility complex (MHC) I and MHC II molecules and co-stimulatory markers including CD80, CD86 and CD40 which enables pDC to present antigens to CD4+ T cell and cross-prime CD8+ T cells.[7]

To study the important role of pDC in immune response and immune disorders, different human leukemic pDC lines e.g. CAL-1, GEN2.2 and PMDC05 with characteristic pDC phenotype and function were generated.[8–10] However, stable cell lines of murine pDC that display characteristics comparable to that of primary pDC are missing. Previously we generated a conditionally immortalized myeloid dendritic cell line derived from murine bone marrow of mice carrying a double ligand (Dexamethasone and Doxycycline) inducible SV40 Large T-Antigen. [11] Here we describe a conditionally immortalized pDC line with the behaviour of primary cells and long term stability in culture which depends only on Doxycycline for induction of SV40 Large T-Antigen.

## Materials and methods

### Ethics statement

Animal experiments were carried out in strict accordance with the German Animal Welfare Act. The protocol was approved by the Committee on the Ethics of the Landesdirektion Dresden, Germany (Permit Number: DD24-5131/354/31).

### Animals

Transgenic Rosa-rtTA mice (ROSA26-STOP-rtTA-IRES-EGFP) [12] were crossed with PGK-Cre mice [13] to remove the STOP cassette upstream the rtTA-IRES-EGFP transgene. Mice carrying tetracycline inducible large T Antigen [14] were intercrossed with the ROSA26-rtTA-IRES-EGFP mice to obtain offspring with a Tetracycline-inducible SV40 large T Antigen (Rosa-rtTA-flox-Tag+). OTI (C57BL/6-Tg(TcraTcrb)1100Mjb/J; transgenic T cell receptor recognizes the OVA peptide 257–264) and OTII (B6.Cg-Tg(TcraTcrb)425Cbn/J; transgenic T cell receptor recognizes the OVA peptide 323–339) were purchased from the Jackson Laboratory (Bar Harbor, USA). For cell or organ isolation mice were sacrificed by cervical dislocation under anesthesia. All mice were bred under pathogen-free conditions in the animal facility of the Technische Universitaet Dresden.

### Preparation of bone marrow cells and generation of plasmacytoid dendritic cells

To generate mouse pDC, bone marrow cells were isolated from a female Rosa-rtTA-flox-Tag+ mouse femur and tibia by flushing the bones with 1× PBS containing 0.5% (w/v) bovine serum albumin (BSA; Sigma Aldrich, Taufkirchen, Germany) and 2 mM EDTA (Sigma Aldrich). After red blood cell lyses with ACK lysis buffer (Thermo Fisher Scientific, Dreieich, Germany) remaining cells were cultured at 37°C in a humidified atmosphere with 5% (v/v) CO_2_ in complete RPMI medium (GE Healthcare, Munich, Germany) supplemented with 10% (v/v) fetal bovine serum (FBS; Thermo Fisher Scientific), 2 mM L-glutamine, 100 IU mL^-1^ penicillin, 100 μg mL^-1^ streptomycin (GE Healthcare), 1 mM sodium pyruvate, 10 mM HEPES (Biochrom, Berlin, Germany) and 50 μM β-mercaptoethanol (Sigma Aldrich). To induce differentiation into pDC, Fms-like tyrosine kinase 3 ligand (Flt3L; 100 ng mL^-1^) produced by a B16 melanoma cell line was added to the culture.[15] To induce large T antigen expression, cells were treated with Doxycycline (Dox; 1 μg mL^-1^; Sigma Aldrich). After 7 days, CD11b+ cells were depleted from the non-adherent fraction using CD11b Microbeads (Miltenyi Biotec, Bergisch Gladbach, Germany). Remaining pDC (CD11b-) were cultured in RPMI complete medium supplemented with Flt3L and Dox.

### Generation of single cell clones

Bone marrow derived, Flt3L-differentiated pDC were counted, centrifuged and adjusted to 2.5 cells mL^-1^ RPMI complete medium supplemented with Flt3L and Dox. Next, these pDC were seeded at 200 μl cell suspension per 96 well cavity, which corresponds to 48 cells per 96 well plate. Plates were monitored microscopically for the appearance of single cell colonies and transferred to larger culture volumes, respectively. Stable growing clones were further characterized.

### Cytochemical cell characterization

For panoptic Pappenheim staining cells were seeded on poly-L-lysin-coated glass slides and incubated with or without TLR9 ligands overnight. Culture medium was removed and slides were first incubated for 3 min with May-Grünwald-solution (Merck, Darmstadt, Germany), followed by incubation with Giemsa working solution consisting of 1 vol Giemsa stock solution and 20 vol buffer solution pH 6.8 (both Merck) for 20 min. Finally, slides were washed with buffer solution pH 6.8 and air-dried. Cells were monitored microscopically (Zeiss Axiovert 200M; Carl Zeiss Microscopy, Jena, Germany).

### Stimulation of dendritic cells and analysis of cytokine secretion

*In vitro* differentiated single cell clones, Dox-pDC and BM-pDC (2×10^6^ ml^-1^) were stimulated in RPMI complete medium supplemented without or with TLR9 ligands ODN CpG1585 (type A, 1 μM) or ODN CpG1826 (type B; 1 μM; both Invivogen, San Diego, USA) for 20 hours. Whereas CpG type A induces strong IFNα response and the maturation of pDC, type B induces the secretion of IL-6 and TNFα, but only less IFNα. Cell culture supernatants were analysed for cytokine secretion with cytometric bead arrays (CBA; IL-6, IL-10, TNFα; BD Biosciences, Heidelberg, Germany) together with LSRII flow cytometer (BD Biosciences) or ELISA (IFNα, IFNβ; Thermo Fisher Scientific). Cytokine levels were normalized to standard curves of recombinant cytokines and in case of CBAs analysed using the FCAP Array software (BD Biosciences), respectively. The amount of secreted cytokines were represented as femtogram (fg) per cell.

### Flow cytometry

Prior to flow cytometry, cells were washed in staining buffer (0.05% (w/v) BSA, 2 mM EDTA in 1× PBS) and treated with FcR blocking reagent (Miltenyi Biotec) for 10 min. Subsequently, *In vitro* differentiated single cell clones and Dox-pDC were stained with the following antibodies for 30 min at 4°C: CD11c-APC, MHC-I-FITC, MHC-II-PE, SiglecH-PE, CD86-PE-Cy7, CD289 (TLR9)-FITC, CD11b-V500, B220-PerCP, CD8α-APC-Cy7 (all BD Biosciences) and CD9-FITC (Thermo Fisher). T lymphocytes were stained with the following antibodies: CD3-FITC, CD4-V500, CD8α-APC-Cy7, CD44-APC and IFNγ-APC-Cy7 (all BD Biosciences); CD62L-PerCP-Cy5.5 and RORγt-PerCP-ef710 (all Thermo Fisher Scientific). Flow cytometry was performed using LSRII and FlowJo analysis software (V10; FlowJo, Ashland, USA).

### Antigen-presentation studies

Dox-pDC were pulsed with Ovalbumin grade V (OVA-V, 100 μg mL^-1^) or low endotoxin Ovalbumin (OVA LE, 100 μg mL^-1^; both Sigma Aldrich) in RPMI complete medium for 16 hours, washed twice with 1× PBS and counted. For *in vivo* immunization, 2.5×10^6^ OVA-V-pulsed Dox-pDC were injected i.p. into CD45.1-C57Bl/6J mice. Fourteen days post transplantation pan T cells were isolated from spleen by magnetic bead separation (Pan T cell isolation kit II; Miltenyi Biotech). For antigen provocation, *in vitro* OVA-V-pulsed Dox-pDC were cocultured with purified pan T cells in a ratio 1:5. Proliferation of CD4+ and CD8+ T cells as well as the frequency of effector memory T cells (TEM) was analysed after 5 days of coculture. Antigen presentation studies using OTI and OTII mice were performed with OVA-LE in combination with TLR9 stimulation. CD4+ and CD8+ T cells were isolated from spleen of OTII (CD4+) and OTI (CD8+) mice by magnetic bead separation (CD4 T cell isolation Kit, CD8 T cell isolation kit II; Miltenyi Biotech). The purity of CD4+ or CD8+ T cells (CD3+) was greater than 97%. Dox-pDC and BM-pDC were pulsed with OVA-LE in the absence or presence of TLR9 ligands CpG A or CpG B. After two hours Dox-pDC were washed and cocultured together with CD4+ or CD8+ T cells in a ratio 1:5. The frequency of activated Th1 (CD4+IFNγ+), Th17 (CD4+RORγt+) and cytotoxic T cells (CD8+IFNγ+) was analysed by LSRII flow cytometer.

### Proliferation, apoptosis and cell cycle analysis

For cell proliferation analysis, 2×10^6^ cells were labelled with 1 μM violet proliferation dye VPD450 (Thermo Fisher Scientific) according to manufacturer instructions and analysed by LSRII flow cytometer. To quantify apoptosis and necrosis, 2×10^6^ cells were stained with Annexin V-PE antibody (BD Biosciences) and Hoechst 33342 (1 μg/ml, Sigma Aldrich) for 15 min and analysed by flow cytometry. Finally, cells were analysed by LSRII flow cytometer.

### Statistics

If not stated otherwise, data were analysed with one- or two-way ANOVA models. The numbers of experimental and technical replicates are shown in the figure legends. P-values of less than 0.05 were considered statistically significant. The statistical analyses were done with GraphPad Prism software (Version 5.04; GraphPad Software, La Jolla, USA).

## Results

### Generation of the immature plasmacytoid dendritic cell line Dox-pDC

**Fig 1:**
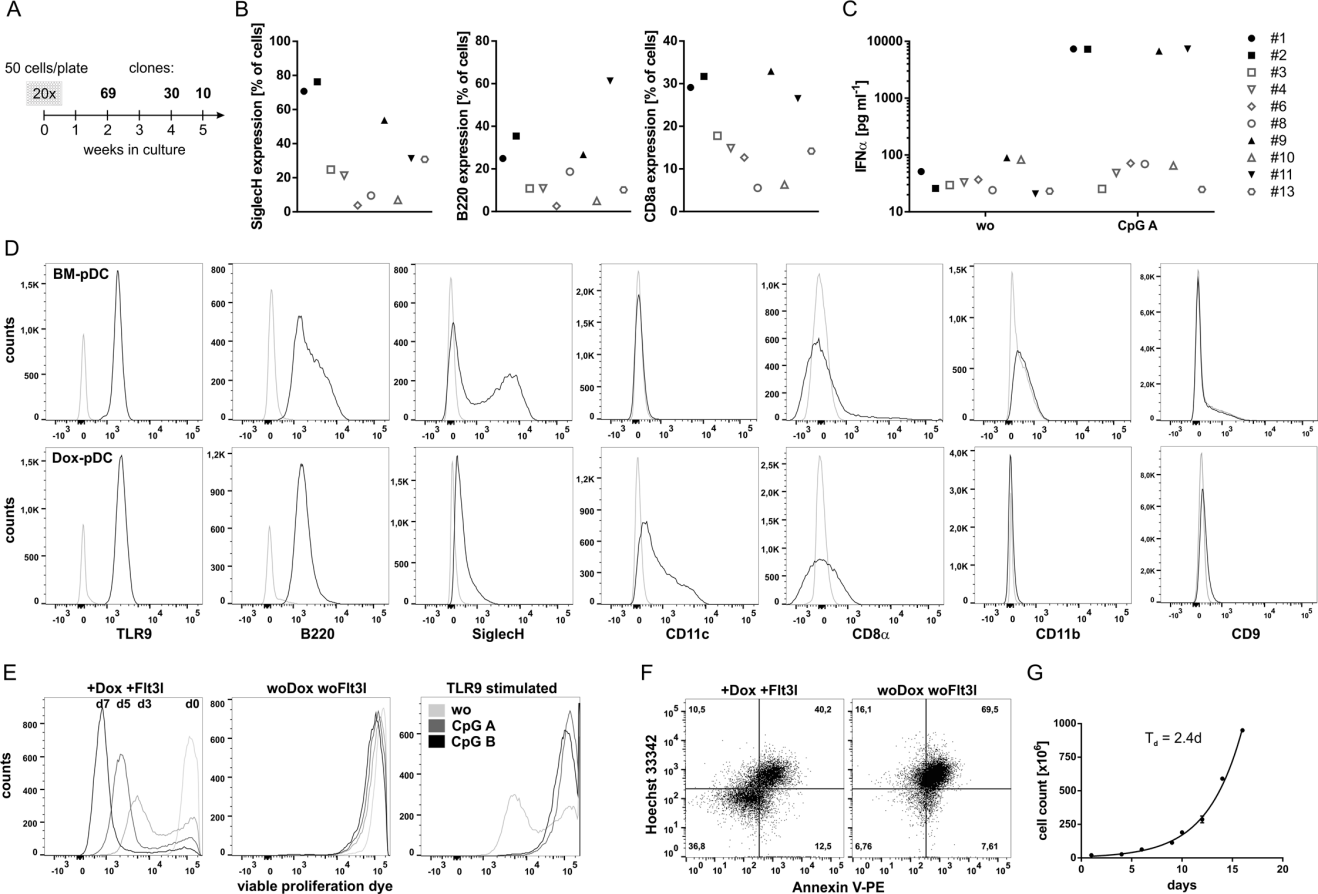
Phenotype of immature Dox-pDC. (A) Scheme of generating Dox-pDC line. (B) Expression of pDC marker by the final 10 single cell clones. (C) IFNα secretion of the final 10 single cell clones. (D) Flow cytometric analysis of BM-pDC and Dox-pDC for different cell-specific markers. (E) Proliferation of Dox-pDC in the presence or absence of Dox and Flt3l and TLR9 activation detected with the viability proliferation dye 450 at day 0, 3, 5, 7 and analysed by flow cytometry. (F) Apoptosis staining of Dox-pDC in the presence or absence of Dox and Flt3l analysed by flow cytometry. (G) Doubling time of Dox-pDC calculated with an exponential growth equation. The figure shows representative results out of 2–4 experiments.

To overcome the limitations on using primary pDC we aimed to generate an immature pDC mouse cell line with a characteristic phenotype of primary mouse cells. To obtain a defined cell population we first generated single cell clones from bone-marrow derived, Flt3L-differentiated pDC (Fig 1A). Out of twenty 96-well plates 69 cell colonies (7% of input) developed within 14 days of culture in the presence of Flt3L and Dox. After two additional weeks 30 of these colonies (3% of input) displayed a stable proliferation and were transferred into 48-well format. After a total of 5 weeks 10 remaining stable single cell clones (1% of input) were further cultured and characterized for typical pDC marker IFNα, SiglecH, B220 and CD8α. Four clones (#1, 2, 9 and 11) expressed the surface molecules SiglecH, B220 and CD8α (Fig 1B) and secrete IFNα upon CpG A-mediated TLR9 stimulation (Fig 1C). In contrast, the other six clones (#3, 4, 6, 8, 10 and 13) showed lower expression of at least one surface molecule (Fig 1B and 1C) and did not secrete IFNα upon CpG A-mediated TLR9 stimulation (Fig 1C). Bone marrow derived pDC (BM-pDC) are characterized by the expression of TLR9, B220, SiglecH, CD8α, low to intermediate CD11c, CD9, PDCA1 and the absence of CD11b (Fig 1D). Based on these data we further investigated the single cell clone #2 (hereinafter named Dox-pDC) that expressed TLR9, B220, SiglecH and CD8α comparable to BM-pDC (Fig 1D). In contrast to *in vitro* differentiated bone marrow derived primary mouse pDC (BM-pDC), the Dox-pDC show an increased expression of CD11c and CD9 (Fig 1D) whereas expression of SiglecH is reduced. Both, BM-pDC and Dox-pDC are negative for CD11b (Fig1 D) and PDCA-1 (data not shown). In the presence of Dox and Flt3L the Dox-pDC demonstrated a constant proliferation (Fig 1E, d3-d7) with a doubling time of 2.4 ± 0.3 days (Fig 1G). In the absence of Dox and Flt3L and after activation with TLR9 ligands CpG A and CpG B the Dox-pDC stopped proliferation and demonstrated an increased apoptosis within 3 days (Fig 1E and 1F).

### Dox-pDC immune profile is comparable to that of bone marrow derived pDC

**Fig 2:**
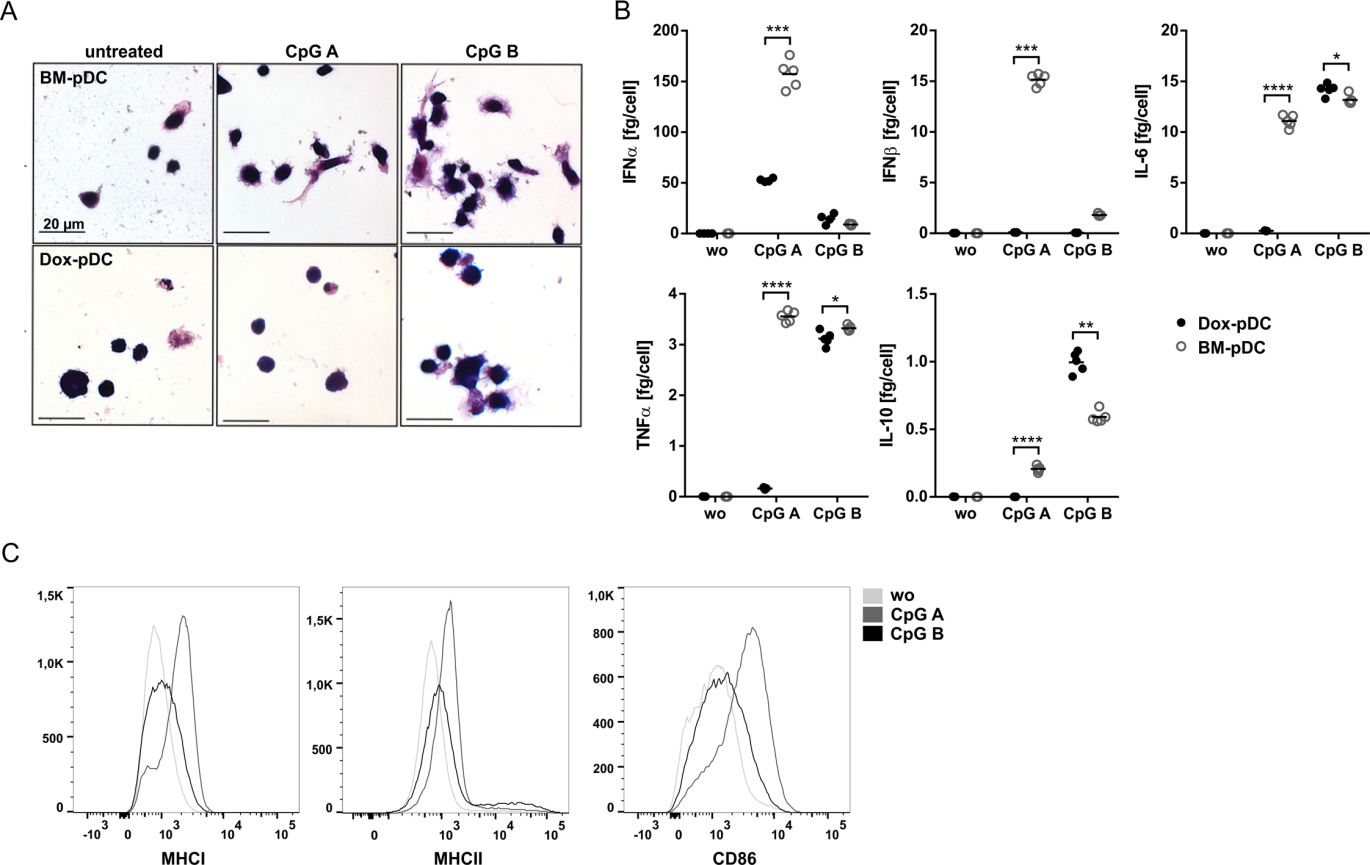
Activation of Dox-pDC. (A) Pappenheim staining of immature (without (wo) CpG A or CpG B) and mature (with CpG A or CpG B) Dox-pDC and BM-pDC. Photomicrographs were taken using a Zeiss Axiovert 200M microscope (magnification: ×630) with a Zeiss AxioCam ICc1. (B) Cytokine secretion of immature and mature Dox-pDC and BM-pDC quantified by ELISA (IFNα, IFNβ) and cytometric bead arrays (IL-6, TNFα and IL-10). (C) Expression of maturation markers (MHCI, MHCII and CD86) in immature and mature Dox-pDC analysed by flow cytometry. Representative results out of 2–3 independent experiments are shown. Statistical significance is indicated, *(P<0.05), **(P<0.01) and ***(P<0.005).

To prove whether Dox-pDC undergo maturation upon contact with pathogens we stimulated the Dox-pDC and BM-pDC with TLR9 ligands CpG A or CpG B. In comparison to non-treated immature Dox-pDC and BM-pDC, TLR9 activated cells exhibited an increased cell size (Fig 2A; CpG B). Since the TLR9 activation of immature pDC results in the secretion of cytokines, we measured the level of IFNα, IFNβ, IL-6, TNFα and IL-10 in the supernatant after 20 hours of cell culture and calculated the amount of cytokines per cell. We detected a strong induction of IFNα in CpG A stimulated Dox-pDC and BM-pDC and a moderate secretion after CpG B stimulation (Fig 2B). Dox-pDC do not secrete IFNβ after TLR9 activation whereas BM-pDC secrete high amounts after CpG A stimulation (Fig 2B). Whereas stimulation with CpG A results in moderate secretion of IL-6, TNFα and no IL-10 in Dox-pDC, CpG B induced a massive production of those cytokines (Fig 2B). In comparison to immature (unstimulated) Dox-pDC, activation with TLR9 ligands results in the upregulation of MHCI, MHCII and the co-stimulatory molecule CD86 with a stronger impact of CpG A (Fig 2C).

### Dox-pDC activate and polarize T cells *in vivo* and *in vitro*

**Fig 3:**
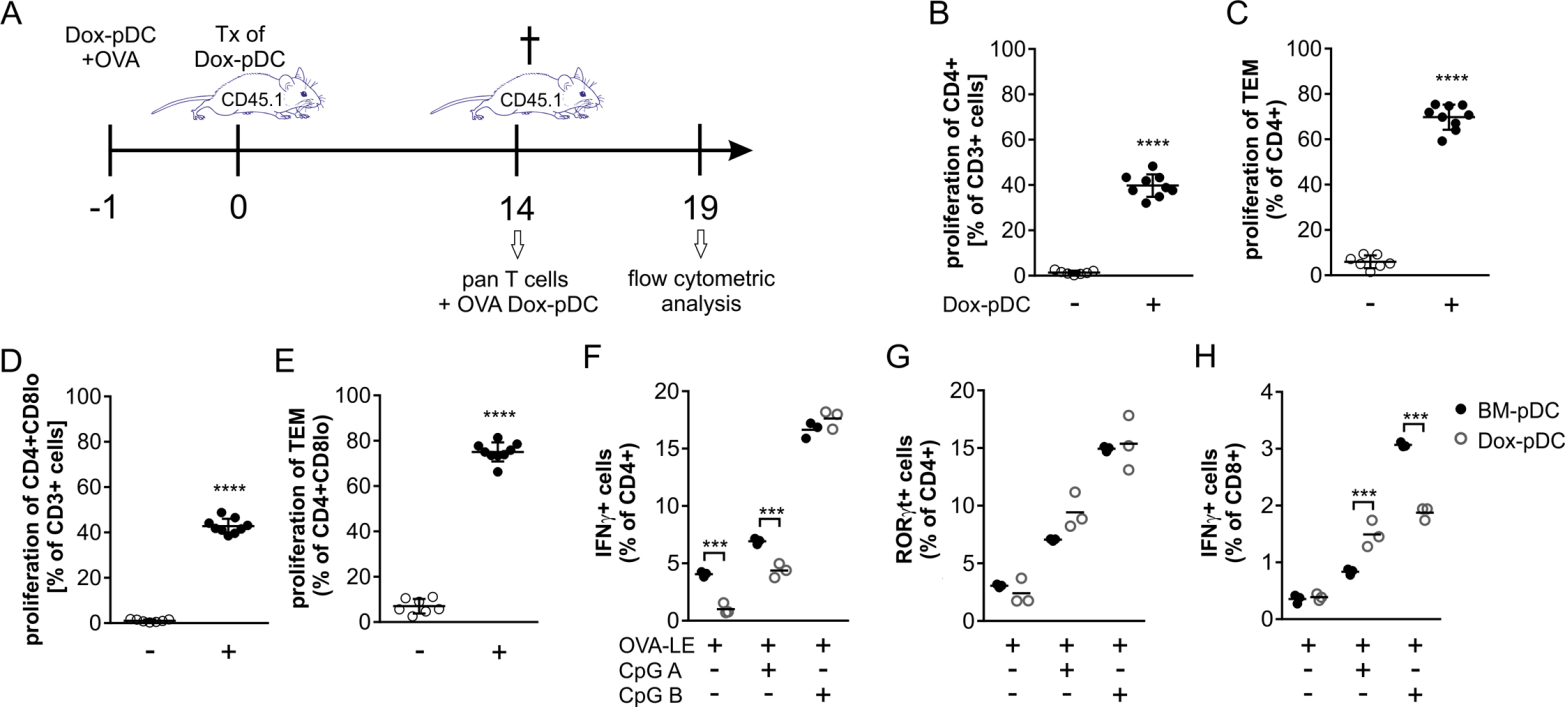
Induction of T cell response. (A) *In vivo* immunization model with OVA-V antigen. (B, C) Proliferation of CD4+ T cells (B) and effector memory T cells (CD4+ TEM; C) activated with OVA-V-pulsed Dox-pDC. (D, E) Proliferation of CD4+CD8lo T cells (D) and effector memory T cells (CD4+CD8lo TEM; E) activated with OVA-V-pulsed Dox-pDC. (F-H) Frequency of Th1 (IFNγ+CD4+; F), Th17 (RORγt+CD4+; G) and CD8+ T cells (IFNγ+CD8+; H) polarized with OVA-LE-pulsed and TLR9-activated Dox-pDC or BM-pDC. Results are expressed as means ± SD from 3–9 mice per group. Statistical significance is indicated, ***(P<0.005) and ****(P<0.001).

Antigen presentation and induction of an adaptive immune response are fundamental functions of pDC. By the use of an Ovalbumin (OVA)-triggered immunization model we tested whether Dox-pDC are able to activate T cells *in vivo* (Fig 3A). We transplanted CD45.1 mice with OVA-V pulsed Dox-pDC. Fourteen days later, we isolated and cocultured T cells from these mice with OVA-V pulsed Dox-pDC *in vitro* and analysed their proliferation. In comparison to CD4+ T cells cultured in the presence of OVA-V without Dox-pDC, CD4+ T cells cocultured together with OVA-V-pulsed Dox-pDC proliferated significantly more (Fig 3B). Furthermore, the frequency of proliferating CD4+ effector memory T cells (TEM; CD62L-CD44+) in cocultures with OVA-V loaded Dox-pDC was also enhanced (Fig 3C). Although we did not detect any proliferation of CD8+ T cells in this model, we detected a strong increase of proliferating CD4+CD8lo T cells compared to T cells incubated with OVA-V alone (Fig 3D). As expected, also the frequency of proliferating effector memory T cells was enhanced in the CD4+CD8lo population (Fig 3E).

To test, whether Dox-pDC in comparison to BM-pDC are able to polarize naïve T cells into T helper subtypes Th1, Th2 and Th17, we stimulated Dox-pDC and BM-pDC with low endotoxin OVA (OVA-LE) in the absence or presence of TLR9 ligands CpG A or CpG B, respectively. Splenic CD4+ T cells from OTII mice cocultured with OVA-LE pulsed Dox-pDC or BM-pDC only contain a small population of IFNγ+ (Th1) or RORγt+ (Th17) population (Fig 3F and 3G). The additional activation of TLR9 on Dox-pDC or BM-pDC with CpG A or CpG B significantly increased the Th1 and Th17 population with a greater influence of CpG B (Fig 3F and 3G). In contrast, we did not detect a Th2 population. Splenic CD8+ T cells from OTI mice showed significantly increased frequency of IFNγ+ cells when cocultured with OVA-LE loaded and TLR9 stimulated Dox-pDC or BM-pDC (Fig 3H). As already observed for the CD4+ T cells CpG B is much more potent in polarizing T cells than CpG A.

## Discussion

Plasmacytoid dendritic cells are a unique dendritic cell subset that primarily promotes an effective antiviral immune response by rapid and massive secretion of IFN. Due to their increasing implication in the pathogenesis of immune-mediated diseases stable and reliable *in vitro* cell systems for e.g. detailed mechanistic investigations are required. Murine primary pDC are mainly isolated from spleen or can be differentiated from bone marrow cells by addition of Flt3L to the cell culture. However, the achieved cell numbers and the stability in culture for longer periods are not satisfying.

Although several human pDC lines are well described they all have a leukaemic origin.[8–10] Whereas the pDC line CAL-1 was generated from a patient with blastic natural killer cell lymphoma, the cell lines GEN2.2 and PMDC05 originated from patients with pDC-derived acute leukaemia. All these cells lines were described to have natural pDC immune function, but mainly due to their leukemic origin they showed differences in terms of their IFNα secretion and their unique pDC marker expression, i.e. BDCA-2 when compared to primary human pDC.[8–10]

In the present study we established a defined murine pDC clone that stably proliferates by the reversible induction of a Doxycycline-inducible SV40 large T Antigen. After withdrawal of Doxycycline the Dox-pDC stopped their proliferation and undergo apoptosis within 3 days (Fig 1). The Dox-pDC expressed the pDC marker TLR9, B220, SiglecH, CD11c and CD8α but not the myeloid marker CD11b (Fig 1). In comparison to Dox-pDC we detected lower CD11c expression in primary bone marrow derived pDC. In contrast to human pDC murine, Flt3L-differentiated pDC generally express CD11c.[16, 17]

Upon contact with pathogens pDC are activated and mature. This process is accompanied with morphological changes, shifted cell cycle distribution, upregulation of MHC and co-stimulatory molecules and secretion of cytokines. We showed that activation of Dox-pDC by the TLR9 ligand CpG A (ODN1585) resulted in the secretion of IL-6 and TNFα and massive amounts of IFNα (Fig 2). Stimulation with TLR9 ligand CpG B (ODN1826) induced high levels of IL-6, TNFα and IL-10 (Fig 2). The opposed effect of CpG A and CpG B especially regarding IFNα and IL-10 may be explained by the regulatory function of IL-10. It has been demonstrated that IL-10 inhibits the IFNα secretion in murine and human pDC.[18, 19] Activation of Dox-pDC by TLR9 stimulation is accompanied by the upregulation of MHCI, MHCII and CD86 (Fig 2). Several studies showed that TLR9 activation results in the maturation of human and murine pDC characterized by upregulation of MHC and costimulatory molecules.[20–23]

Presentation of antigens to naïve T cells and thus their activation and polarization is an additional and important function of pDC.[24] Ovalbumin-loaded Dox-pDC were able to activate T cells *in vivo* and *in vitro* (Fig 3). They induced the proliferation of CD4+ T cells, but not CD8+ cells when pulsed with OVA alone. The capability of pDC to cross-prime CD8+ T cells is dependent on the activation of TLR9.[25] Simultaneous activation of OVA-loaded Dox-pDC with CpG A or CpG B resulted not only in the activation of IFNγ+ CD8+ T cells but also in the polarization of CD4+ Th1 and Th17 cells (Fig 3). We did not detect any T cells of the subtype Th2 which can be explained by the concentration of OVA we used. Boonstra et al. showed that OVA-loaded and TLR9-activated BM-derived pDC do not induce a Th2 phenotype at moderate to high concentration of antigen and induce only a weak Th2 polarization with low dose of antigen.[16] In general the Dox-pDC are able to induce a Th1/Th17 phenotype when loaded with the model antigen OVA.

Our novel murine Dox-pDC cell line with its long-term stability is easy to culture and comprise the unique characteristics of primary pDC. With its great potential for investigating pDC-mediated effects *in vitro* and *in vivo*, Dox-pDC pose an essential tool for elucidating the role of pDC in health and disease.

## References

[1] Liu YJ. IPC: professional type 1 interferon-producing cells and plasmacytoid dendritic cell precursors. Annu Rev Immunol. 2005;23:275–306. doi: 10.1146/annurev.immunol.23.021704.115633 . 15771572 10.1146/annurev.immunol.23.021704.115633

[2] Gilliet M, Cao W, Liu YJ. Plasmacytoid dendritic cells: sensing nucleic acids in viral infection and autoimmune diseases. Nat Rev Immunol. 2008;8(8):594–606. doi: 10.1038/nri2358 . 18641647 10.1038/nri2358

[3] Reizis B, Bunin A, Ghosh HS, Lewis KL, Sisirak V. Plasmacytoid dendritic cells: recent progress and open questions. Annu Rev Immunol. 2011;29:163–83. doi: 10.1146/annurev-immunol-031210-101345 ; PubMed Central PMCID: PMCPMC4160806. 21219184 10.1146/annurev-immunol-031210-101345 PMC4160806

[4] Romagnani C, Della Chiesa M, Kohler S, Moewes B, Radbruch A, Moretta L, et al Activation of human NK cells by plasmacytoid dendritic cells and its modulation by CD4+ T helper cells and CD4+ CD25hi T regulatory cells. Eur J Immunol. 2005;35(8):2452–8. doi: 10.1002/eji.200526069 . 15997468 10.1002/eji.200526069

[5] Gerosa F, Gobbi A, Zorzi P, Burg S, Briere F, Carra G, et al The reciprocal interaction of NK cells with plasmacytoid or myeloid dendritic cells profoundly affects innate resistance functions. J Immunol. 2005;174(2):727–34. . 15634892 10.4049/jimmunol.174.2.727

[6] Santini SM, Lapenta C, Logozzi M, Parlato S, Spada M, Di Pucchio T, et al Type I interferon as a powerful adjuvant for monocyte-derived dendritic cell development and activity in vitro and in Hu-PBL-SCID mice. J Exp Med. 2000;191(10):1777–88. ; PubMed Central PMCID: PMCPMC2193160. 10811870 10.1084/jem.191.10.1777 PMC2193160

[7] Swiecki M, Colonna M. The multifaceted biology of plasmacytoid dendritic cells. Nat Rev Immunol. 2015;15(8):471–85. doi: 10.1038/nri3865 ; PubMed Central PMCID: PMCPMC4808588. 26160613 10.1038/nri3865 PMC4808588

[8] Chaperot L, Blum A, Manches O, Lui G, Angel J, Molens JP, et al Virus or TLR agonists induce TRAIL-mediated cytotoxic activity of plasmacytoid dendritic cells. J Immunol. 2006;176(1):248–55. . 16365416 10.4049/jimmunol.176.1.248

[9] Maeda T, Murata K, Fukushima T, Sugahara K, Tsuruda K, Anami M, et al A novel plasmacytoid dendritic cell line, CAL-1, established from a patient with blastic natural killer cell lymphoma. Int J Hematol. 2005;81(2):148–54. . 15765784 10.1532/ijh97.04116

[10] Narita M, Watanabe N, Yamahira A, Hashimoto S, Tochiki N, Saitoh A, et al A leukemic plasmacytoid dendritic cell line, PMDC05, with the ability to secrete IFN-alpha by stimulation via Toll-like receptors and present antigens to naive T cells. Leuk Res. 2009;33(9):1224–32. doi: 10.1016/j.leukres.2009.03.047 . 19443030 10.1016/j.leukres.2009.03.047

[11] Richter C, Thieme S, Bandola J, Laugsch M, Anastassiadis K, Brenner S. Generation of inducible immortalized dendritic cells with proper immune function in vitro and in vivo. PLoS One. 2013;8(4):e62621 doi: 10.1371/journal.pone.0062621 ; PubMed Central PMCID: PMCPMC3633827. 23626840 10.1371/journal.pone.0062621 PMC3633827

[12] Belteki G, Haigh J, Kabacs N, Haigh K, Sison K, Costantini F, et al Conditional and inducible transgene expression in mice through the combinatorial use of Cre-mediated recombination and tetracycline induction. Nucleic Acids Res. 2005;33(5):e51 doi: 10.1093/nar/gni051 ; PubMed Central PMCID: PMCPMC1069131. 15784609 10.1093/nar/gni051 PMC1069131

[13] Lallemand Y, Luria V, Haffner-Krausz R, Lonai P. Maternally expressed PGK-Cre transgene as a tool for early and uniform activation of the Cre site-specific recombinase. Transgenic Res. 1998;7(2):105–12. . 9608738 10.1023/a:1008868325009

[14] Anastassiadis K, Rostovskaya M, Lubitz S, Weidlich S, Stewart AF. Precise conditional immortalization of mouse cells using tetracycline-regulated SV40 large T-antigen. Genesis. 2010;48(4):220–32. doi: 10.1002/dvg.20605 . 20146354 10.1002/dvg.20605

[15] Mach N, Gillessen S, Wilson SB, Sheehan C, Mihm M, Dranoff G. Differences in dendritic cells stimulated in vivo by tumors engineered to secrete granulocyte-macrophage colony-stimulating factor or Flt3-ligand. Cancer Res. 2000;60(12):3239–46. . 10866317

[16] Boonstra A, Asselin-Paturel C, Gilliet M, Crain C, Trinchieri G, Liu YJ, et al Flexibility of mouse classical and plasmacytoid-derived dendritic cells in directing T helper type 1 and 2 cell development: dependency on antigen dose and differential toll-like receptor ligation. J Exp Med. 2003;197(1):101–9. doi: 10.1084/jem.20021908 ; PubMed Central PMCID: PMCPMC2193804. 12515817 10.1084/jem.20021908 PMC2193804

[17] Brawand P, Fitzpatrick DR, Greenfield BW, Brasel K, Maliszewski CR, De Smedt T. Murine plasmacytoid pre-dendritic cells generated from Flt3 ligand-supplemented bone marrow cultures are immature APCs. J Immunol. 2002;169(12):6711–9. . 12471102 10.4049/jimmunol.169.12.6711

[18] Contractor N, Louten J, Kim L, Biron CA, Kelsall BL. Cutting edge: Peyer's patch plasmacytoid dendritic cells (pDCs) produce low levels of type I interferons: possible role for IL-10, TGFbeta, and prostaglandin E2 in conditioning a unique mucosal pDC phenotype. J Immunol. 2007;179(5):2690–4. . 17709480 10.4049/jimmunol.179.5.2690

[19] Duramad O, Fearon KL, Chan JH, Kanzler H, Marshall JD, Coffman RL, et al IL-10 regulates plasmacytoid dendritic cell response to CpG-containing immunostimulatory sequences. Blood. 2003;102(13):4487–92. doi: 10.1182/blood-2003-07-2465 . 12946990 10.1182/blood-2003-07-2465

[20] Kerkmann M, Rothenfusser S, Hornung V, Towarowski A, Wagner M, Sarris A, et al Activation with CpG-A and CpG-B oligonucleotides reveals two distinct regulatory pathways of type I IFN synthesis in human plasmacytoid dendritic cells. J Immunol. 2003;170(9):4465–74. . 12707322 10.4049/jimmunol.170.9.4465

[21] Bauer M, Redecke V, Ellwart JW, Scherer B, Kremer JP, Wagner H, et al Bacterial CpG-DNA triggers activation and maturation of human CD11c-, CD123+ dendritic cells. J Immunol. 2001;166(8):5000–7. . 11290780 10.4049/jimmunol.166.8.5000

[22] Krug A, Towarowski A, Britsch S, Rothenfusser S, Hornung V, Bals R, et al Toll-like receptor expression reveals CpG DNA as a unique microbial stimulus for plasmacytoid dendritic cells which synergizes with CD40 ligand to induce high amounts of IL-12. Eur J Immunol. 2001;31(10):3026–37. doi: 10.1002/1521-4141(2001010)31:10<3026::AID-IMMU3026>3.0.CO;2-H . 11592079 10.1002/1521-4141(2001010)31:10<3026::aid-immu3026>3.0.co;2-h

[23] Liu YC, Gray RC, Hardy GA, Kuchtey J, Abbott DW, Emancipator SN, et al CpG-B oligodeoxynucleotides inhibit TLR-dependent and -independent induction of type I IFN in dendritic cells. J Immunol. 2010;184(7):3367–76. doi: 10.4049/jimmunol.0903079 ; PubMed Central PMCID: PMCPMC2892962. 20181884 10.4049/jimmunol.0903079 PMC2892962

[24] Villadangos JA, Young L. Antigen-presentation properties of plasmacytoid dendritic cells. Immunity. 2008;29(3):352–61. doi: 10.1016/j.immuni.2008.09.002 . 18799143 10.1016/j.immuni.2008.09.002

[25] Mouries J, Moron G, Schlecht G, Escriou N, Dadaglio G, Leclerc C. Plasmacytoid dendritic cells efficiently cross-prime naive T cells in vivo after TLR activation. Blood. 2008;112(9):3713–22. doi: 10.1182/blood-2008-03-146290 ; PubMed Central PMCID: PMCPMC2572799. 18698004 10.1182/blood-2008-03-146290 PMC2572799

